# Treatise on Diseases of Women

**Published:** 1889-07

**Authors:** 


### Book Reviews.

---

TREATISE ON THE DISEASES OF WOMEN. By Alexander J. C. Skene, M. D., Professor of Gynecology in the Long Island College Hospital, etc., New York. D. Appleton and Company, 1888.

In the preface of the volume before us it is stated that "this book was written for the purpose of bringing together the fully matured and essential facts in the science and art of gynecology." No higher praise is needed than to say that the purpose has been successfully carried out. As an exposition of the present status of gynecology, the work has no superior to-day. The author evidently has no hobby to warp his views and blunt his powers of observation, and in this respect the work differs from the majority of the treatises on gynecology which have heretofore appeared.

A marked feature of Skene's work is a notable conservatism in the discussion of all subjects. The tendency of modern gynecology is to run to extremes of opinion, and it is as refreshing as it is unusual to find a work in which this tendency is conspicuously absent. This moderation is especially noticeable in the discussion of the use of pessaries, the treatment of uterine flexions and the pathology of pelvic cellulitis, subjects upon which the majority of gynecologists hold very radical views.

In treating uterine fibroids, the author gives full and complete description of the treatment of these neoplasms by electrolysis. A short treatise upon electro-physics furnishes ample information to enable a practitioner of average intelligence to use this therapeutic agent intelligently and satisfactorily.

One-third of the volume is devoted to a consideration of diseases of the bladder and urethra, a subject which receives only a cursory notice in most works on gynecology. This portion is mainly a reproduction of the author's well-known and popular monograph. Until very recently it constituted the only treatise on this subject in the English language and still remains the best.

The illustrative plates which are numerous and mostly original, are the finest which have ever appeared in any work on gynecology. Unlike many illustrations which we have seen, they are a positive help to the understanding of the text. The plates, for instance, which represent the operation of trachelorraphy are so perfect that a study of them would render superfluous the printed description of the operation.

Taken all in all this work is the best one at the present time upon the subject of which it treats. We feel no hesitation therefore in recommending it to any one who may desire a text book either for study or for reference. V. O. H.

